# Lifetime glucocorticoid profiles in baleen of right whale calves: potential relationships to chronic stress of repeated wounding by Kelp Gulls

**DOI:** 10.1093/conphys/coy045

**Published:** 2018-08-20

**Authors:** Alejandro A Fernández Ajó, Kathleen E Hunt, Marcela Uhart, Victoria Rowntree, Mariano Sironi, Carina F Marón, Matias Di Martino, C Loren Buck

**Affiliations:** 1 Center for Bioengineering Innovation, Department of Biological Sciences, Northern Arizona University, 617 S. Beaver St., Flagstaff, AZ, USA; 2 Instituto de Conservación de Ballenas, Capital Federal, O’Higgins 4380, Ciudad Autónoma de Buenos Aires, Argentina; 3 Southern Right Whale Health Monitoring Program, Los Alerces 3376, Puerto Madryn, Chubut, Argentina; 4 School of Veterinary Medicine, University of California, 1089 Veterinary Medicine Drive Ground Floor West, Davis, CA, USA; 5 Department of Biology, University of Utah, 257 South 1400 East University of Utah, Salt Lake City, UT, USA; 6 Ocean Alliance/Whale Conservation Institute, 32 Horton St, Gloucester, MA, USA; 7 Diversidad Biológica IV, Universidad Nacional de Córdoba, Av. Vélez Sársfield 299, Córdoba, Argentina

**Keywords:** Baleen, calves, corticosterone, cortisol, stress, validations

## Abstract

Baleen tissue accumulates stress hormones (glucocorticoids, GC) as it grows, along with other adrenal, gonadal and thyroid hormones. The hormones are deposited in a linear fashion such that a single plate of baleen allows retrospective assessment and evaluation of long-term trends in the whales’ physiological condition. In whale calves, a single piece of baleen contains hormones deposited across the lifespan of the animal, with the tip of the baleen representing prenatally grown baleen. This suggests that baleen recovered from stranded carcasses of whale calves could be used to examine lifetime patterns of stress physiology. Here we report lifetime profiles of cortisol and corticosterone in baleen of a North Atlantic right whale (‘NARW’—*Eubalaena glacialis*) calf that died from a vessel strike, as well as four southern right whale (‘SRW’—*Eubalaena australis*) calves that were found dead with varying severity of chronic wounding from Kelp Gull (*Larus dominicanus*) attacks. In all five calves, prenatally grown baleen exhibited a distinctive profile of elevated glucocorticoids that declined shortly before birth, similar to GC profiles reported from baleen of pregnant females. After birth, GC profiles in calf baleen corresponded with the degree of wounding. The NARW calf and two SRW calves with no or few gull wounds had relatively low and constant GC content throughout life, while two SRW calves with high numbers of gull wounds had pronounced elevations in baleen GC content in postnatal baleen followed by a precipitous decline shortly before death, a profile suggestive of prolonged chronic stress. Baleen samples may present a promising and valuable tool for defining the baseline physiology of whale calves and may prove useful for addressing conservation-relevant questions such as distinguishing acute from chronic stress and, potentially, determining cause of death.

## Teaser

Baleen from stranded carcasses of five right whale calves (four southern and one North Atlantic) contain cortisol and corticosterone patterns that may represent longitudinal profiles of stress exposure over time. Calves with high wounding from Kelp Gulls had markedly higher glucocorticoids in postnatal baleen than did less wounded and unwounded calves.

## Introduction

Understanding the impacts of natural and anthropogenic disturbances on the behaviour and physiology of wildlife is critical to predicting resiliencies and vulnerabilities to environmental change. The habits, body size and longevity of large whales present numerous logistical complications that have severely challenged collection of data needed to elucidate basic natural history parameters as well as impacts of natural and anthropogenic disturbances on their population (Meyer-Gutbord and Greene, 2017). For example, methods for live capture or blood sample collection from free-swimming large whales have not been developed, and thus key life history traits such as gestation length, inter-calving interval and reproductive rate have only been estimated from resighting records. Little is known of the responses of individual whales to environmental change, including potential impacts of natural or anthropogenic stressors on health, mortality risk and future reproduction.

In most vertebrates, stressful stimuli (e.g. injury, storms, starvation) cause elevations in secretion of adrenal glucocorticoids (‘GCs’, cortisol and/or corticosterone), which then elicit a variety of physiological and behavioural responses that presumably help the animals to deal with the stressor (McEwen and Wingfield, 2003; French *et al.*, 2007; Romero *et al.*, 2009; Meylan *et al.*, 2010). Prolonged exposure to chronic stress, however, can exceed the animal’s ability to cope with the stressors and thus negatively impact body condition, health, future reproduction and survival. Indeed, the GCs themselves, if elevated for prolonged periods, can directly inhibit immune function, growth and reproduction. In cases of prolonged chronic stress, GCs may ultimately decline from their initially elevated state and may even fall below normal baseline levels, a phenomenon sometimes referred to as ‘adrenal exhaustion’ (Romero and Wingfield, 2016). Ultimately, glucocorticoid-based parameters are increasingly used as indicators of the occurrence, severity and cumulative effects of stressors on wildlife. A large body of literature now exists linking glucocorticoid measures to acute and chronic stress in both terrestrial and marine wildlife (Haukenes and Buck, 2006; Fridinger *et al.*, 2007; Brewer *et al.*, 2008a, b; Williams *et al.*, 2008; Hunt *et al.*, 2013; Romero and Wingfield, 2016). Yet, little information exists on patterns of glucocorticoids with repeated or chronic stress in mysticete whales, largely because whales are remarkably difficult to access and study.

In recent years, researchers have developed novel techniques to fill this knowledge gap for the large whales. Endocrine analyses of alternative (non-plasma) sample types have enabled assessment of glucocorticoids in live whales through minimally invasive approaches that do not require lethal sampling. Some of these sample types provide information on only a single point in time or a brief 1–2 days period at most; e.g. faecal samples (Hunt *et al.*, 2006; Rolland *et al.*, 2005, 2017), respiratory vapour samples (Hogg *et al.*, 2009; Hunt *et al.*, 2013; Apprill *et al*., 2017; Burgess *et al.*, 2018) and blubber samples (Kellar *et al.*, 2013; Vu *et al.*, 2015). Though such studies have proven highly useful given the extreme dearth of endocrine information from large whales, repeated sampling of the same individual over time is typically rare and long-term impacts of stress have remained difficult to assess (Hunt *et al.*, 2013). Photography based approaches (visual health assessment, photogrammetry) presently have the best ‘sampling rate’ and thus the greatest likelihood of repeated information from single individuals across time (Hunt *et al.*, 2013; Durban *et al.*, 2015, 2016; Christiansen *et al.*, 2018), but even in the most intensively monitored populations there are typically large gaps (months to years) in individual photographic records. Earplug analysis has potential to recover the entire lifespan of the whale, via analysis of annually deposited layers of cerumen (earwax) in earplugs recovered from stranded carcasses (Trumble *et al.*, 2013), but temporal resolution of cerumen layers is relatively coarse (6–12 months increments). Additionally, earplugs are only rarely collected due to the logistical challenge of intact excision from deep within a large cetacean skull, and for calves in particular are seldom found.

Recently Hunt *et al.* (2014, 2016, 2017a, b) validated methods for longitudinal studies of glucocorticoids through the analysis of whale baleen, a tissue which, like mammalian hair, contains progressive time series of hormones that are continuously deposited in growing baleen. Baleen is the filter-feeding apparatus of the mysticete whales, and consists of long fringed plates of stratified, cornified tissue (hairs embedded in a firm calcium–salt matrix) that grow continuously and slowly downward from the upper jaw (Hunt *et al.*, 2014). Baleen is readily accessible at necropsy and is routinely collected from strandings, providing a unique opportunity for retrospective longitudinal analyses of hormone patterns from archived specimens.

Concentrations of cortisol and corticosterone from single pieces (plates) of whale baleen indicate that baleen likely records a continuous ‘timeline’ of adrenal activity spanning the time-period of baleen growth, which is several years in most species (Hunt *et al.*, 2016, 2017a; ms in prep.). In North Atlantic right whales (*Eubalaena glacialis*, ‘NARW’), and bowhead whales (*Balaena mysticetus*), GCs are elevated in regions of baleen known to have been grown during documented stressors, such as chronic entanglements in fishing gear or known periods of chronic disease (Lysiak *et al.*, 2018; Hunt *et al.*, 2016, 2017a; ms in prep.). Baleen GCs are also elevated during energetically demanding reproductive states (pregnancy, lactation) that are associated with high GCs in other sample types (Rolland *et al.*, 2005; Hunt *et al.*, 2017a, b). Interestingly, baleen cortisol and baleen corticosterone may show quite different patterns, raising the question of which GC may be more informative to measure (Hunt *et al.*, 2017a). Mysticete whales have traditionally been considered a ‘cortisol-dominant’ taxon, i.e. with higher concentrations of cortisol than of corticosterone, but this assumption is supported primarily by samples collected during acute-stress situations (e.g. hunting, live-stranding) that may not necessarily represent baseline patterns. In baleen from adult whales, corticosterone tends to be present in whale baleen in higher concentration than cortisol, and is often more readily detectable in small samples (Hunt *et al.*, 2017a, b). However, some cases indicate that both GCs tend to elevate in near-synchrony during periods of severe stress (e.g. chronic entanglement in fishing gear; Lysiak *et al.*, 2018). Current data indicate that study of both hormones, when possible, may provide a more complete picture of adrenal physiology than assaying either hormone alone (Hunt *et al.*, 2017a).

Baleen GCs may be especially informative in whale calves (whales <1 year old), in which a single baleen plate likely captures the lifetime history of the animal, given that the tip of the baleen does not erode away until the second year of life (Lubetkin *et al.*, 2008). Stable isotope data indicate that in calves, the tip of the baleen represents the second half of gestation, i.e. the tip was grown prenatally (Lubetkin *et al.*, 2008). The timing of birth is easily distinguished by a visible ‘natal notch’ or groove at the boundary between prenatally grown baleen and post-natally grown baleen. Thus, quantification of hormones extracted from baleen offers an unparalleled opportunity for detailed retrospective analysis of patterns in stress hormones over the calf’s entire life, including part of prenatal development.

As a first step in assessing utility of baleen glucocorticoid analysis for assessment of stress in whale calves, we sought to compare baleen glucocorticoid patterns in chronically wounded calves to those of less-wounded and unwounded calves, i.e. representing a continuum of exposure to presumed chronic stress. For this analysis we employed baleen specimens from two species of right whales, the southern right whale (*Eubalaena australis*, ‘SRW’) and the closely related NARW. SRW was selected for this study because the population that calves off Península Valdés, Argentina, has suffered extremely high calf mortality in recent years. Since 2003, at least 706 calves died at Valdés, including a peak of 113 deaths in 2012 (53.2/year 2003–17) (Sironi *et al.*, 2015; Di Martino *et al.*, 2018), greatly surpassing the average of 8 calves per year in previous decades (1993–2002) (Rowntree *et al.*, 2013). This rate of calf mortality is higher than any levels ever reported for baleen whales (Thomas *et al.*, 2013; Di Martino *et al.*, 2018). At this calving location, Kelp Gulls (*Larus dominicanus*) have developed a unique parasitic behaviour of landing on the backs of the whales (in particular whale calves) and feeding on their skin and blubber, creating large open wounds on their backs (Rowntree *et al.*, 1998; Thomas *et al.*, 2013; Marón *et al.*, 2015). Behavioural changes in gull-harassed whales are well documented (Sironi *et al.*, 2009), yet a clear link between gull harassment and physiological/health impacts has not yet been established. During necropsies of SRW calf carcasses at Valdés, baleen is routinely collected, and animals are scored for presence and extent of cutaneous wounds including the number and extent of the distinctive lesions made by Kelp Gull attacks. This has allowed for the comparison of baleen GC patterns in minimally wounded vs. severely wounded calves, as well as the changes in patterns in GCs across the calf’s months of life (i.e. as it approached death). The closely related NARW, in contrast, are not harassed by gulls but are frequently struck by ships (Meyer-Gutbrod and Greene, 2017), events that often result in rapid death. Recently we obtained a baleen plate from a NARW calf that had been sighted repeatedly in good condition before its death, when it was killed within minutes by multiple propeller strikes from a large vessel. This specimen offers a valuable comparison point of a calf that can be assumed not to have been subjected to chronic stress.

In this study we aimed to determine glucocorticoid patterns typical of right whales subjected to the chronic stress of repeated wounding (SRW calves with high numbers of gull wounds) vs. those with little or no evidence of chronic stress (SRW calves with few/zero wounds, and the ship-struck NARW calf). Our specific goals were to: (i) validate cortisol and corticosterone assays for the quantification of both GCs in SRW baleen, a species not yet studied using this method and (ii) assess lifetime GC profiles across the full length of baleen from right whale calves with varying levels of wounding, comparing highly wounded whales to minimally wounded and unwounded whales. Using baleen GC concentration as a proxy for physiological stress, we hypothesized that homoeostasis of whale calves is adversely affected by gull attacks, and we predicted that concentration of baleen GCs would positively correlate with the severity of gull induced wounding, would increase over time in highly wounded SRW calves, and would remain low in the ship-struck NARW and SRW calves with few or zero lesions (i.e. gull wounds).

## Materials and methods

### Baleen samples

SRW baleen samples were recovered from necropsies of stranded southern right whale calf carcasses at Península Valdés-Argentina by the Southern Right Whale Health Monitoring Program (SRWHMP) team. From 2003 to 2010 SRWHMP sample database, we identified four calves that were larger 6 m in length and that had contrasting levels of gull wounding (Table 1). Calves larger than 6 m include post-natal growth on their baleen (opposite to neonatal calves, <5 m, in which the baleen can consist entirely of pre-natally grown tissue). Birth date of these SRW calves is not known and baleen growth rate in SRW calves is not definitively known but estimates of age from snout-fluke body length indicates all SRW calves in this study were likely less than ~1–2 months old at the time of death (McAloose *et al.*, 2016). The presence, number and size of gull-wound lesions on the dead SRW calves had been previously scored using methods of Marón *et al.* (2015). Briefly, photographs taken during necropsies were quantified and each lesion was assigned to one of seven objectively defined size categories: extra-small (XS), small (S), medium (M), large (L), extra-large (XL), double XL (XXL) and triple XL (XXXL). To compare relative gull wounding among animals, size and number of lesions were converted to the number of XS-lesion-equivalents. For example, a whale with one XS lesion and one M lesion (number of lesions = 2) was considered to have a total area of five XS lesions; one M lesion is roughly equivalent in area to four XS lesions (Marón *et al.*, 2015). All dead calves were found fresh and without major skin damage, i.e. minimal detached skin or damage by scavengers. Cause of death is not conclusively known for any of the SRW calves chosen for the present study (i.e. no evidence of human interaction, predation).

**Table 1:** Study animal details

Species	Whale ID	Gender	Size (m)	Gull lesions	Baleen length (cm)	Sampling points
SRW	071610PV-Ea03	M	6.46	0	19.5	18
SRW	102905PV-Ea28	F	6.78	4	26	24
SRW	091109PV-Ea45	F	6.8	51	22	20
SRW	091208PV-Ea49	Unk	6.95	39	24.5	15^a^
NARW	Eg #4681	M	9.02^b^	0	44	43

SRW = southern right whale; NARW = North Atlantic right whale; gull lesions = number of small-lesion equivalents, from Marón *et al.* (2015). ^a^091208PV-Ea49 pre-natal section was sampled every 2 cm. ^b^Eg #4681 length was likely impacted by the multiple propeller strikes wounds, and total length is suspected overestimated for this animal (Niemeyer, Misty 2018 com. pers.).

A single NARW baleen specimen was recovered by IFAW (International Fund for Animal Welfare) during the 2016 necropsy of a male NARW calf, Eg #4681 (previously identified as ‘2016Calfof#1281’, carcass accession #D-IFAW16-082Eg). As a critically endangered species, NARW are under intensive observation and most individuals have a repeated sightings history; furthermore, individual NARW are uniquely identifiable via individually distinctive patterns of scars and of callosities on the head, or, in calves, close association with an identified mother (Hamilton *et al.*, 2007). Sightings records from the North Atlantic Right Whale Sightings and Identifications Databases (www.rwcatalog.neaq.org) indicate that calf Eg #4681 was first sighted on 12 January 2016; due to size and appearance of the calf on this date (i.e. lack of foetal skin folds, no cyamids on tail flukes), date of birth is estimated at ~1–2 months before its initial sighting. Calf Eg #4681 was sighted 34 more times during its life, always accompanied by its mother (Eg #1281) and was noted to be in good body condition. It was last sighted alive on 28 April 2016 in Cape Cod Bay, at which point both calf and mother appeared to be in good health. On 5 May 2016, calf Eg #4681 was found floating dead off the shore of Cape Cod, MA, USA. It was towed to shore and necropsied on 6 May 2016. Necropsy findings indicated the cause of death to be vessel strike, with multiple propeller strikes penetrating deeply into the thoracic and abdominal cavities; one propeller strike had bisected the skull. Lack of healing, patterns of haemorrhage and catastrophic trauma to critical organs indicated a near-instantaneous death (i.e. within a few minutes of the vessel strike). There were no indications at necropsy of prior injury, disease or interactions with fishing gear. Estimated age at death was ~5–7 months (P. Hamilton, pers. comm.).

### Preparation and steroid extraction of baleen

#### Cleaning and drying

Baleen samples recovered from necropsies were stored either dry at room temperature or frozen at −20°C until prepared for hormone extraction. Prior studies indicate steroid hormones are stable in baleen tissue for at least 3 decades at room temperature (Hunt *et al.*, 2017a, b). When baleen plates are collected during necropsy, often some gum and other soft tissues remain attached to the plate at the proximal end. To remove all soft tissue from the plates, individual samples were submerged in fresh water for 4 days. Rehydration softens all but the baleen tissue and thus facilitates removal of the soft tissue with a metallic scraper or scalpel. Cleaned plates were then freeze-dried under vacuum in a LabConco Stoppering Tray Dryer lyophilizer (LabConco, Kansas City, MO, USA) until pressure reading of the lyophilizer stabilized for at least 12 h (i.e. indicating that samples were dry). Following drying, samples were stored at room temperature in individual sealed plastic bags along with a 50 g silica gel desiccant pack (Arbor Assays, Ann Arbor, MI, USA) until pulverization for final hormone extraction.

#### Pulverization of baleen tissue

**Figure 1:**
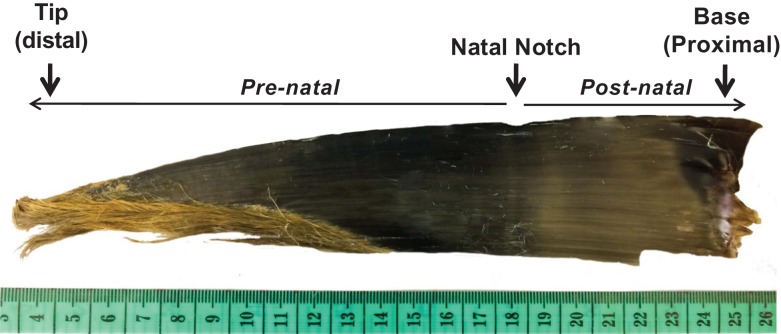
Southern right whale calf baleen plate showing the ‘natal notch’, indicative of the time of birth, the pre- and post-natally growth sections and the proximal and distal tips of the baleen plate.

Based on protocols tested for bowhead whales (*Balaena mysticetus*) and NARW (Hunt *et al.*, 2014, 2016, 2017a, b), all but the distal and proximal tips of the calf baleen plates were pulverized with a hand-held electric rotary grinder (Dremel model 395 type 5) fitted with a tungsten ball-tip and flexible extension. The Dremel was used to abrade a series of ~0.5 cm-wide grooves across the posterior face of each plate at 1 cm intervals from base to tip (except for Calf 091208PV-Ea49, for which the pre-natal section was sampled every 2 cm). Baleen growth rate in adult right whales averages 15 days per 1 cm of baleen (Rowntree *et al.*, 2008; Lysiak, 2018; Hunt *et al.*, 2016), but is typically faster in juvenile whales than in adults (e.g. bowhead whales, Lubetkin *et al.*, 2008; and SRW, Rowntree *et al.*, 2008). Baleen growth rates of calves have never been determined for any species. Therefore, though the 1 cm sampling intervals employed here can be tentatively interpreted as representing time intervals about a few days to a week per cm, due to the uncertainty about baleen growth rate of calves we present our data in terms of sampling distance from natal notch. Location on the plate of each drilling location was measured as cm above the natal notch, with prenatally grown baleen represented by negative numbers (i.e. ‘−4 cm’ signifies prenatally grown baleen 4 cm below the natal notch; see Fig. 1). Each groove started at the labial edge of the plate and extended 1–2 cm transverse to the baleen length, with powdered baleen samples collected on a piece of weighing paper below the baleen. After drilling, 20 mg of the powder was weighed on a digital scale accurate to within ±0.0001 g. To reduce potential effects of static electric charge on handling and weighing of baleen powder samples, a work station ionizer (SPI No. 94000, SPIwesstek.com) was placed inside the hood by the scale and was always activated whenever baleen powder was handled. Considerable care was taken to avoid cross-contamination by shielding other regions of the plate with adhesive tape during drilling, as well as comprehensive cleaning between samples, as follows: after every sample was weighed, the entire baleen plate, all equipment and the fume hood were cleaned with compressed air blow (i.e. blowing any baleen powder particles into the exhaust vent of the hood) followed by thorough rinsing of the work surface, baleen, digital scale, weighing spatulas and Dremel tips with 70% ethanol, with multiple changes of gloves. Weighed samples were placed in a 16 × 100 mm^2^ borosilicate glass tube and capped securely until extraction within 72 h of drilling.

The distal-most part of a baleen plate is usually loose hairs, and the most proximal portion is a hollow and thin tissue; in the small baleen plates of calves, neither the distal or proximal sampling areas are amenable to pulverization with the Dremel tool. Therefore, these samples (very distal-most sample and very proximal-most sample) from each plate were excised and pulverized in a bead beater (Mini-Beadbeater-24, Bio Spec Products Inc., Bartlesville, OK, USA). Weighing then proceeded as described above.

#### Hormone extraction

Following our previous studies (Hunt *et al.*, 2014, 2016, 2017a, b), hormones were extracted from weighed samples with 100% methanol. Our prior studies on large adult plates used 100 mg samples of powder per sampling location, but the small calf baleen plates generate insufficient powder to routinely use 100 mg samples. We therefore used a sample mass of 20 mg baleen powder extracted with 1.6 mL methanol (i.e. 80:1 ratio of mL of solvent to g of sample), after pilot testing revealed that this sample mass and solvent:sample ratio yields good detectability for both glucocorticoids in SRW and NARW calf baleen, with acceptably low variation (i.e. <20% combined inter-extract variation and inter-assay variation from subsamples of a single powder pool extracted separately; Fernández Ajó, unpublished data). Once methanol was added, tubes were vortexed for 2 h at room temperature (Large Capacity Mixer, Glas-Col, Terre Haute, IN, USA; speed set on 40), and centrifuged for 1 min at 4025 g, after which supernatant was transferred to a 13 × 100 mm^2^ borosilicate tube and dried at 35°C for a minimum of 4 h in a sample evaporator (Speedvac 121P, Waltham, MA) under vacuum. Dried samples were reconstituted in 0.50 ml assay buffer (×065 buffer; Arbor Assays, Ann Arbor, MI, USA), sonicated for 5 min, vortexed for 5 min, transferred to 1.5 mL vapour proof o-ring-capped cryovials, stored overnight at −80°C and decanted to a new cryovial to remove any remaining baleen particulates. This was considered the ‘1:1’ (full-strength, neat) extract. All samples were frozen at −80°C until assay within 2 months. The total number of samples from all calves combined was 120 (Table 1).

#### Hormone assays and validation

Commercial enzyme immunoassay (EIA) kits (Arbor Assays kit corticosterone #K014 and cortisol #K003, Ann Arbor, MI, USA) were used to quantify immunoreactive corticosterone and cortisol in calf baleen extracts. The two assays had previously been validated for NARW baleen extract (Hunt *et al.*, 2017a) but not yet for SRW baleen extract, so we conducted tests of parallelism and accuracy using standard methods described in Grotjan and Keel (1996). To test for parallelism, a pool of SRW baleen extract was serially diluted in assay buffer to produce eight dilutions (range 1:1–1:128). All dilutions were assayed as unknowns in both the cortisol and corticosterone EIAs, following which the linear portions of the two binding curves in each assay were compared for equality of slope (i.e. serially diluted pool vs. known-concentration hormone standards). Parallelism of these two binding curves within a given assay indicates that the assay antibody binds well to an immunoreactive component in the sample of interest, with very similar affinity as to pure parent hormone; this is considered strong evidence that the hormone is in fact present in the sample (Grotjan and Keel, 1996). Assay accuracy (aka ‘matrix effect test’, ‘interference test’) was assessed by spiking a full standard curve with pooled 1:1 SRW baleen extract and assaying alongside a second standard curve that was spiked only with assay buffer. The resulting graph of apparent total hormone concentration vs. known standard concentration was assessed for linearity and slope; a slope within the range of 0.7–1.3 (ideal slope = 1.0) indicates the assay correctly discriminates low-dose from high-dose samples without interference from sample matrix (i.e. baleen powder components).

Following validations, all samples were assayed at 1:1 for both hormones. Assays followed standard QA/QC criteria including a full standard curve, NSB (non-specific binding) and zero dose (‘blank’) in every EIA microplate, with assay of all NSBs, zeros, standards and unknowns in duplicate. Any sample that exceeded 10% coefficient of variation between duplicates was re-analysed. Both hormone assays included one extra ‘low’ standard, produced by mixing equal volumes of the lowest standard and assay buffer. Intra-assay and inter-assay variations for all assays were <10%. For antibody cross-reactivities, assay sensitivities and other methodological details, see Hunt *et al.* (2017a).

### Statistical analysis

Parallelism results for cortisol and corticosterone were plotted as percentage of antibody bound vs. log[concentration]. An *F* test was employed to assess differences between slopes of the linear portions of the resulting binding curves for serially diluted SRW baleen pool and the standard curve of each assay. Accuracy results were plotted as apparent total concentration (i.e. standard + SRW baleen pool) vs. known standard concentration and assessed by linear regression, with acceptable accuracy defined as *r*^2^ ≥ 0.98 and slope within 0.7–1.3. To assess whether the two glucocorticoids yielded similar results, we performed correlation tests to compare cortisol vs. corticosterone concentrations. *F* tests, linear regressions and correlations all used two-tailed tests with Prism 7.0c for Macintosh; descriptive statistics (means and coefficients of variation within treatments) were calculated with Microsoft Excel. Data are plotted as means ± SEM and all differences were considered significant at *P* < 0.05.

## Results

### EIA validations

**Figure 2:**
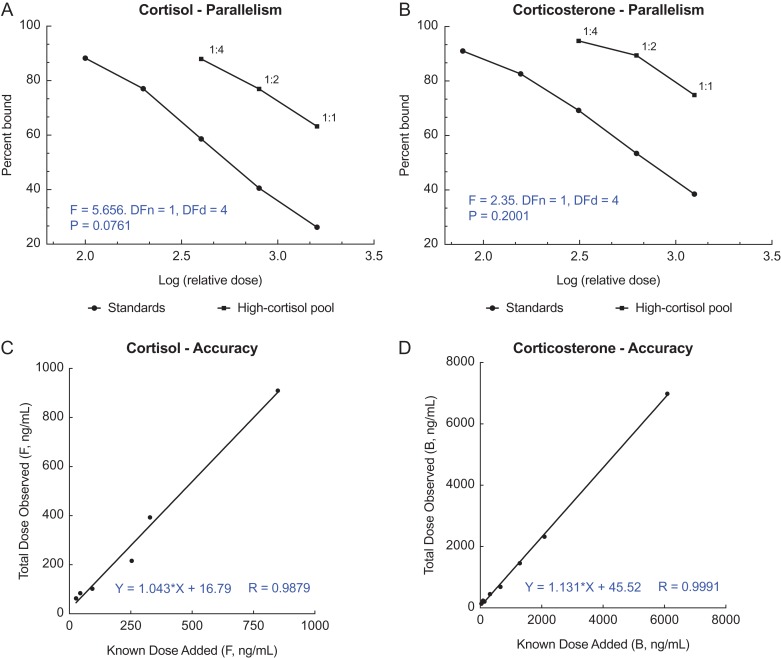
Parallelism (**A** and **B**) and accuracy results (**C** and **D**) for cortisol and corticosterone enzyme immunoassays tested with pooled southern right whale baleen extract. Parallelism results (A and B) do not include dilutions above 1:4 that had non-detectable hormone; statistical results from *F* test slope comparison are shown in blue. Accuracy (C and D) was tested with 1:1 extract; best-fit regression equation is shown in blue.

Serially diluted SRW baleen samples yielded displacement curves parallel to the respective standard curves, with no significant differences in slope for either the cortisol (*F*_(1,4)_ = 5.66; *P* = 0.08) or corticosterone (*F*_(1,4)_ = 2.35; *P* = 0.20) (Fig. 2, top panels; A and B). Only the three most concentrated dilutions (1:1, 1:2 and 1:4) yielded detectable glucocorticoids in the tests of parallelism. Accuracy was acceptable for both assays of SRW baleen extract, as indicated by a linear relationship between observed and expected hormone concentration (*r*^2^ ≥ 0.98), and a slope within the desired range of 0.7–1.3 (cortisol slope = 1.04; corticosterone slope = 1.13; Fig. 2, bottom panels; C and D).

### Evaluation of longitudinal trends in stress hormones

In general, both GCs were detectable along the full length of the baleen plates in all individuals. Cortisol and corticosterone significantly correlated with each other for each individual plate (071610PV-Ea03: *r* = 0.66, *P* = 0.0029, *n* = 18; 102905PV-Ea28: *r* = 0.92, *P* < 0.0001, *n* = 24; 091109PV-Ea45: *r* = 0.96, *P* < 0.0001, *n* = 20; 091208PV-Ea49: *r* = 0.99, *P* < 0.0001, *n* = 15; Eg #4681: *r* = 0.69, *P* < 0.0001, *n* = 43).

**Figure 3:**
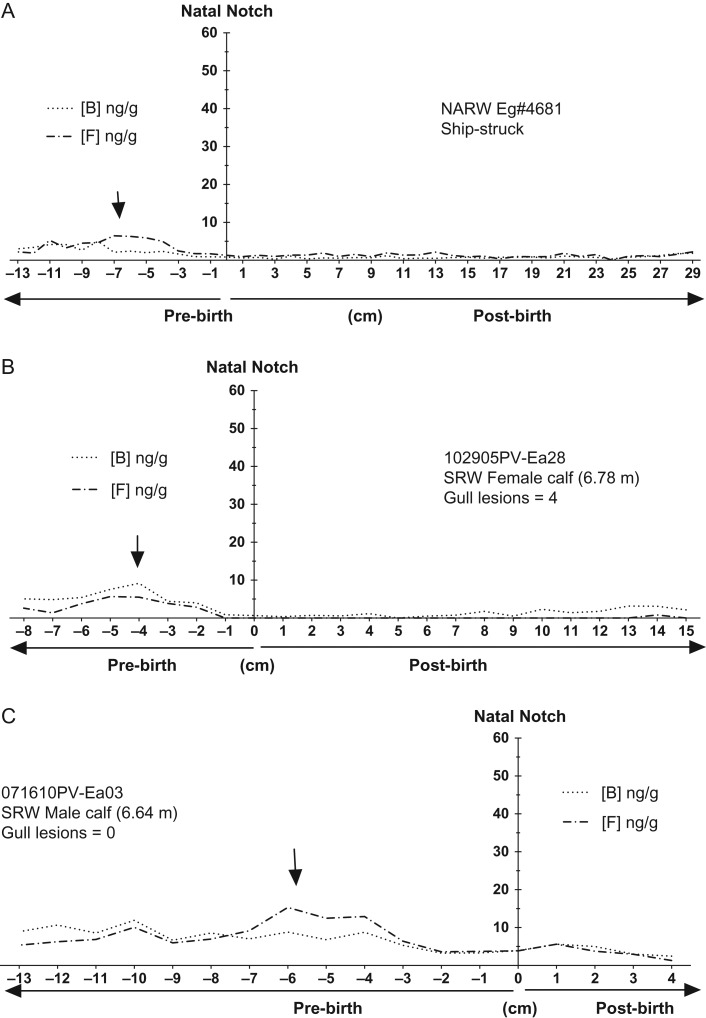
Immunoreactive cortisol (dashed line) and corticosterone (dotted line) across the full length of baleen plates from right whale calves with low or no evidence of chronic wounding: (**A**) NARW, male calf ‘Eg #4681’; (**B**) SRW female calf ‘102905PV-Ea28’; and (**C**) SRW male calf ‘071610PV-Ea03’. The *Y* axis is centred at the ‘natal notch’ (zero on the *X* axis) indicating birth. *Y* axis is scaled to 60 ng/g to allow comparisons with the highly wounded calves (Fig. 4). *X*-axis indicates cm above the natal notch, i.e. positive values along the *X*-axis denote post-natal baleen growth whereas negative values indicate pre-natal growth. Arrows indicate ‘knolls’, regions with relatively higher elevations in glucocorticoids which could be associated with maternal GCs for the late pregnancy stages. In figure legend, B = corticosterone and F = cortisol.

**Figure 4:**
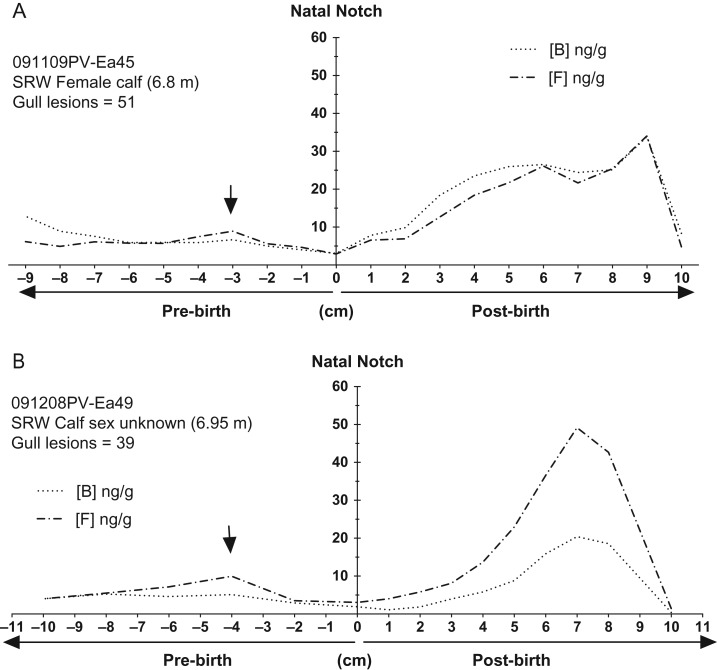
Immune reactive cortisol (black dots and solid lines) and corticosterone (black dots) across the full length of baleen plates from two calves with extensive cutaneous wounding from Kelp Gulls: (**A**) SRW female calf ‘091109PV-Ea45’ and (**B**) SRW calf of unknown sex ‘091208PV-Ea49’. The *Y*-axis is centred at the ‘natal notch’ (zero on the *X* axis) indicating the time of birth. *X*-axis indicates cm above the natal notch, i.e. positive values along the *X*-axis denote post-natal baleen growth while negative values indicate pre-natal growth. Arrows indicate ‘knolls’, regions with relatively higher elevations in glucocorticoids which could be associated with maternal GCs for the late pregnancy stages. In figure legend, B = corticosterone and F = cortisol.

Glucocorticoid concentrations of prenatally grown baleen (i.e. tip of plate below the natal notch) were elevated in all whales (as compared to presumed baselines just after birth) for both GCs, with maximal prenatal concentrations generally in the −6 to −4 cm region. Prenatal GC concentrations were lower near the natal notch (i.e. near the time of birth) in all whales (Figs 3 and 4, black arrows) as compared to earlier in the gestational record, distal from the natal notch.

After birth, whales with few or no gull inflicted lesions had relative low and nearly invariable GC concentrations for the entire length of the plate, with the ship-struck NARW calf generally exhibiting the ‘flattest’ (i.e. no obvious peaks) profile of GC content compared to the SRW calves (<10 ng/g; Fig. 3). In contrast, GC concentrations in the baleen of the two heavily wounded whales increased markedly after birth and attained maximum concentrations one or two cm before the gum line, a region of the baleen corresponding to fairly recently grown baleen (Fig. 4). Furthermore, in the two heavily wounded calves, concentrations of glucocorticoids precipitously declined to very low levels in the most recently grown region of the baleen sampled at the gum line (Fig. 4).

## Discussion

Though sample sizes are small in this study (five whales), the longitudinal profiles across the full length of these calf baleen plates suggest that GC concentrations in baleen may indeed represent a lifetime retrospective record of circulating adrenal hormones in right whale calves. Both GCs were detectable, and both presented a variable but positive level of correlation within individuals. Elevated GCs in prenatally grown baleen correspond with our prior findings that GCs are elevated in baleen of pregnant NARW females during the second half of gestation (Hunt *et al.*, 2017a) and with findings of similar patterns in other sample types from pregnant females (e.g. faeces; Hunt *et al.*, 2006). The origin of the prenatal GCs is not known but likely reflects both circulating maternal GCs delivered to the calf via the placenta as well as GCs secreted by the developing calf. In baleen occurring above the natal notch (more recent growth), patterns of baleen GCs corresponded to the severity of gull wounds. Post-birth baleen GC content was highest in calves with many wounds, lower in calves with few or no wounds, and least in the NARW calf that died due to confirmed acute trauma. No acute-trauma cases were available from SRW calves meeting our study criteria of >6 m body length; therefore, the NARW was included as the only known acute-death reference case. It is possible that these two species differ in their hormone deposition rates in baleen, such that GC concentrations in baleen may not be directly comparable across species. However, pilot studies on adult baleen indicate generally comparable baleen glucocorticoid content in adults of at least eight mysticete species, even across distantly related cetacean families (Hunt *et al.*, 2017b; Hunt unpublished data). As NARW and SRW are very closely related, in the absence of evidence to the contrary it appears possible that relative patterns, and perhaps even the absolute GC concentrations, of NARW and SRW calf baleen are comparable to each other. However, additional study of any future calf specimens with evidence of chronic stress (for NARW) or of acute-death (for SRW) would be illuminating. We cannot rule out the possibility of transference of maternal GCs to calves via ingested milk; it is conceivable that the postnatal GC patterns presented here represent some combination of maternal and calf adrenal hormones. However, the sharp decline in GCs in the heavily wounded calves just before death suggests that the calf baleen GC profile likely represents the calf’s physiological state and not the physiological state of the mother transferred to the calf through milk, since mothers are typically less affected by gull wounds than are calves (Marón *et al.*, 2015).

Intriguingly, the two calves with highest wounding exhibited a distinctive ‘rise and fall’ pattern of GC content over time, with baleen GC content increasing and then declining to minimal levels (i.e. below apparent baselines) shortly before death. Similar declines in GCs have been noted in adult whales nearing death from chronic illness (Hunt, ms in prep.) as well as in other cases of prolonged chronic stress such as capture and transference to captivity in birds (Dickens *et al.*, 2009), prolonged severe disease in humans (Marik and Zaloga, 2002; Marik, 2007; Peeters *et al.*, 2017) and, in dolphins, prolonged poor health following exposure to petrochemical contaminants (Scwacke *et al.*, 2014). Controlled experiments of stress physiology in both the laboratory and the field have found that declines in GC can often occur under prolonged or repeated exposure to chronic stressors (Rich and Romero, 2005; Dickens and Romero, 2013). The mechanism of this decline of GC secretion varies, including cases of failure of normal negative feedback mechanisms of the HPA axis, failure of the adrenal gland to respond normally to pituitary stimulation, and/or pathology of the adrenal gland in a moribund individual (Romero and Wingfield, 2016). Regardless of the mechanism, collapse of GC secretion over time from above-normal levels to below-normal levels has often been interpreted to represent an end stage of chronic stress, i.e. failure to cope physiologically with the ongoing stressor (Rich and Romero, 2005; Romero and Wingfield, 2016; Peeters *et al.*, 2017).

## Conclusions

In sum, baleen steroid analysis shows great potential for retrospective assessment of patterns of physiological stress. Our findings suggest that the longitudinal patterns in GC concentration in right whale calves closely correspond to the degree of chronic physical wounding, i.e. severity of Kelp Gull lesions in SRW calves. Moreover, pre-natally grown baleen may reflect changes in hormonal condition from maternal origin. Calf baleen may thus present a promising and valuable tool for defining baseline physiology of whale calves, an age class for which almost no physiological data has historically been available. Finally, the close concordance between degree of gull wounding with a distinctive rise-and-fall GC pattern—a pattern suggestive of adrenal fatigue or adrenal exhaustion due to chronic stress—suggests that Kelp Gull wounding may in fact be a causal factor in the deaths of at least some SRW calves in Península Valdés. These findings additionally suggest that baleen glucocorticoid analysis may be a fruitful method with which to explore the physiological and population impacts of other types of stressors as well, both natural and anthropogenic, particularly given the potential of baleen analysis to illuminate physiological changes across time. Given recent calls for improved methodology for assessing cumulative impacts of stress in whales (National Academies of Sciences, Engineering and Medicine, 2016), glucocorticoid analyses of baleen collected from stranded specimens may ultimately be able to help discriminate cases of acute vs. chronic stress, may in some cases help resolve potential causes of death, and ultimately may help inform population management, policy decisions and conservation of the large whales.
